# Characterization of an Artificial Swine-Origin Influenza Virus with the Same Gene Combination as H1N1/2009 Virus: A Genesis Clue of Pandemic Strain

**DOI:** 10.1371/journal.pone.0022091

**Published:** 2011-07-25

**Authors:** Xueli Zhao, Yipeng Sun, Juan Pu, Lihong Fan, Weimin Shi, Yanxin Hu, Jun Yang, Qi Xu, Jingjing Wang, Dongjun Hou, Guangpeng Ma, Jinhua Liu

**Affiliations:** 1 Key Laboratory of Zoonosis of Ministry of Agriculture, College of Veterinary Medicine, China Agricultural University, Beijing, China; 2 China Rural Technology Development Center, Beijing, China; 3 The Shandong Animal Disease Control Center, Jinan, Shandong, China; Centers for Disease Control and Prevention, United States of America

## Abstract

Pandemic H1N1/2009 influenza virus, derived from a reassortment of avian, human, and swine influenza viruses, possesses a unique gene segment combination that had not been detected previously in animal and human populations. Whether such a gene combination could result in the pathogenicity and transmission as H1N1/2009 virus remains unclear. In the present study, we used reverse genetics to construct a reassortant virus (rH1N1) with the same gene combination as H1N1/2009 virus (NA and M genes from a Eurasian avian-like H1N1 swine virus and another six genes from a North American triple-reassortant H1N2 swine virus). Characterization of rH1N1 in mice showed that this virus had higher replicability and pathogenicity than those of the seasonal human H1N1 and Eurasian avian-like swine H1N1 viruses, but was similar to the H1N1/2009 and triple-reassortant H1N2 viruses. Experiments performed on guinea pigs showed that rH1N1 was not transmissible, whereas pandemic H1N1/2009 displayed efficient transmissibility. To further determine which gene segment played a key role in transmissibility, we constructed a series of reassortants derived from rH1N1 and H1N1/2009 viruses. Direct contact transmission studies demonstrated that the HA and NS genes contributed to the transmission of H1N1/2009 virus. Second, the HA gene of H1N1/2009 virus, when combined with the H1N1/2009 NA gene, conferred efficient contact transmission among guinea pigs. The present results reveal that not only gene segment reassortment but also amino acid mutation were needed for the generation of the pandemic influenza virus.

## Introduction

Pandemic H1N1/2009 influenza virus spread by human-to-human transmission across the globe at an unprecedented rate after it was first detected in humans [1], [2]. By August 2010, H1N1/2009 virus had spread to more than 215 countries with over 18,000 deaths reported worldwide [3]. The H1N1 influenza virus is now considered to be post-pandemic, yet it is feared that it could still pose a public health threat, especially if it recombines with other influenza viruses, yielding a novel potential pandemic strain [4], [5]. Therefore, the incidence of pandemic H1N1/2009 reiterated the importance of elucidating the genesis of novel emerging pathogens for its prevention and control.

Genetic analyses revealed that the pandemic H1N1/2009 influenza viruses possessed a unique combination of gene segments from swine, human and avian origins; containing PB2 and PA genes of North American avian virus origin, the PB1 gene of human H3N2 virus origin, HA, NP and NS genes of classical swine H1N1 virus origin, and NA and M genes of Eurasian avian-like swine virus origin [6], [7]. These genes have been already established in the triple-reassortant H1N2 and H1N1 swine viruses in North America and in the Eurasian avian-like swine H1N1 viruses for more than 10 years [8]–[11]. Thus, H1N1/2009 was probably a result of an immediate reassortment between two or more swine viruses, i.e. triple-reassortant H1N2 (or H1N1) and the Eurasian avian-like H1N1 swine viruses [7], [12]–[14].

Although the genome of H1N1/2009 virus was described putatively to be of swine origin, a virus with the same gene combination as H1N1/2009 virus has not been reported in swine or other hosts [1], [15] because of poor surveillance of swine influenza viruses worldwide [6]. However, evolutionary analysis indicated that the reassortment between North American and Eurasian swine viruses may not have occurred recently, and it is possible that reassortant virus of this gene combination has been cryptically circulating for years before outbreak in humans [15]. Hence, the virus might have experienced two steps: 1) gene segment reassortment; 2) mutual variation of the eight gene segments under unspecified conditions. The following questions remain: (i) what are the biological characteristics of a virus with the same gene combination as H1N1/2009 virus, and (ii) which gene segment(s) contributes to the different phenotypes between this virus and H1N1/2009 virus?

In the present study, we used reverse genetics to generate a reassortant virus (rH1N1) with the same gene combination H1N1/2009 virus, in which NA and M gene segments were from a Eurasian avian-like H1N1 swine influenza virus and the other six genes were from a triple-reassortant H1N2 swine influenza virus. The rH1N1 virus was characterized *in vitro* and *in vivo* and compared with pandemic H1N1/2009 virus, a North American triple-reassortant swine H1N2 virus, a Eurasian avian-like swine virus, and a seasonal human H1N1 virus. Moreover, the gene segments responsible for the difference of transmissibility between rH1N1 and H1N1/2009 viruses were investigated.

## Results

### Generation and in vitro characteristics of rH1N1 virus

Our previous study found that the NA and M genes of a Eurasian avian-like H1N1 swine influenza virus, A/swine/Fujian/204/2007 (Eurasian-204, accession numbers: FJ536810-FJ536817) [16] and the other six genes of a triple-reassortant H1N2 swine influenza virus A/swine/Guangdong/1222/2006 (Triple-1222, accession numbers: GU086078-GU086085) [8] belonged to the same cluster of H1N1/2009 viruses in the phylogenetic tree. Here, using reverse genetics, we generated the reassortant virus rH1N1 possessing the same gene combination as H1N1/2009 virus with NA and M gene segments from the Eurasian-204 virus, and the other six genes from the Triple-1222 virus. The sequence homology between the rH1N1 and H1N1/2009 influenza strains are shown in Table 1.

**Table 1:** Gene homology of the rH1N1 vs. H1N1/2009 influenza virusesa^a^.

Virus	Subtype	Lineage	Gene segment							
			PB2	PB1	PA	HA	NP	NA	M	NS
A/Mexico/4115/2009	H1N1	H1N1/2009	95.2	95.2	94.6	94.1	95.5	90.9	95.4	93.7
A/California/04/2009	H1N1	H1N1/2009	95.2	95.1	94.6	93.9	95.5	90.8	95.4	93.7
A/Beijing/7/2009 (pBJ09)	H1N1	H1N1/2009	95.0	95.0	94.5	93.8	95.4	90.7	95.6	93.3

a Comparison of the full-length gene sequences.

**Figure 1:**
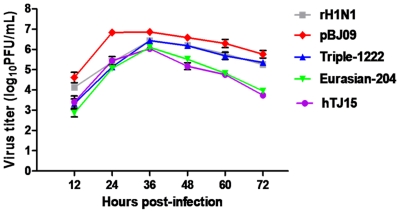
Viral growth kinetics in MDCK cells. MDCK cells were infected with virus at a MOI of 0.05, supernatants were harvested every 12 h, and samples were titrated by plaque assay in MDCK cells. Results represent the average of three independent experiments.

The replication of rH1N1 virus in MDCK cells was tested and compared with those of pBJ09 (accession numbers: HQ533879, HQ533877, HQ533874, HQ533864, HQ533871, HQ533868, HQ533866, and HQ533872), Triple-1222, Eurasian-204, and A/Tianjin/15/2009 (accession numbers: HQ533878, HQ533876, HQ533875, HQ533865, HQ533870, HQ533869, HQ533867, and HQ533873) viruses. MDCK cells were inoculated with each H1 virus at an MOI of 0.05, and the supernatant was titrated at 12, 24, 36, 48, 60, and 72 h p.i. As shown in Fig. 1, there were no significant differences in the viral yields between rH1N1 and pBJ09 viruses (*P*>0.05), except for the titers at 24 h p.i., wherein rH1N1 virus had lower viral yields when compared with pBJ09 virus at each time point (*P*<0.01). Eurasian-204 and hTJ15 viruses replicated with lower titers than pBJ09 virus during the observed period (*P*<0.01).

### Infectivity and pathogenicity of rH1N1 virus in mice

**Figure 2:**
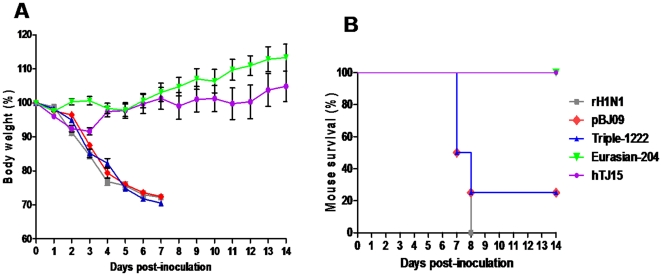
Weight change (*A*) and survival (*B*) of mice. Groups of four BALB/c mice were intranasally inoculated with 10^4.0^ PFU of each virus. Body weight and survival status were checked daily. Mice were euthanized upon the loss of 30% of their initial body weight.

rH1N1, pBJ09, Triple-1222, Eurasian-204, and hTJ15 viruses were intranasally inoculated in mice, respectively. Clinical signs, MID_50_, LD_50_, virus replication, and tissue distribution in mice were examined. As shown in Fig. 2, rH1N1, pBJ09, and Triple-1222 viruses caused sustained weight loss and could cause mice dead. The body weight of the mice infected by Eurasian-204 and hTJ15 viruses increased after slight weight loss and were significantly higher than those of rH1N1, pBJ09, and Triple-1222 viruses since day 5 p.i. (P<0.05), and these mice were with 100% survival rates. Moreover, obvious clinical signs of disease were observed in mice infected by rH1N1, pBJ09, and Triple-1222 viruses, including decreased activity, huddling, hunched posture, and ruffled fur, while mice infected with Eurasian-204 and hTJ15 viruses did not display obvious clinical signs during the course of the experiment. The rH1N1 (MID_50_ = 10^0.5^ PFU), pBJ09 (MID_50_ = 10^0.6^ PFU), and Triple-1222 (MID_50_ = 10^0.3^ PFU) viruses were more infectious in mice than the Eurasian-204 (MID_50_ = 10^1.5^ PFU) and hTJ05 (MID_50_ = 10^1.0^ PFU) viruses. The rH1N1 (LD_50_ = 10^3.0^ PFU), pBJ09 (LD_50_ = 10^3.6^ PFU), and Triple-1222 (LD_50_ = 10^3.5^ PFU) viruses were also more virulent than Eurasian-204 (LD_50_>10^6.0^ PFU) and hTJ05 (LD_50_>10^6.0^ PFU) viruses.

**Figure 3:**
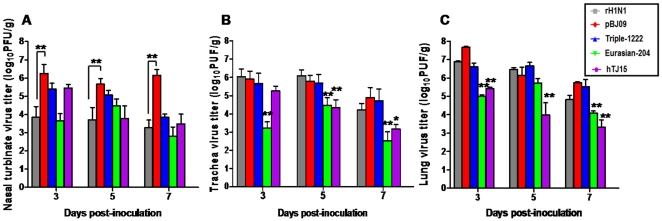
Viral replication in nasal turbinates (*A*), trachea (*B*) and lung (*C*) of mice. Groups of three BALB/c mice were intranasally inoculated with 10^4.0^ PFU of each virus. Titers of virus recovered from the supernatant of homogenized nasal turbinates, trachea and lung on days 3, 5 and 7 p.i. are shown. Results represent the average titers from the tissues of three infected mice per group. The limit of virus detection was 100 PFU/g. ** in Fig. 3*A* indicates the significant difference between rH1N1 and pBJ09 viruses (*P*<0.01; ANOVA). * and ** in Fig. 3*B* and *C* indicates the replication titers of Eurasian-204 or hTJ15 are significantly different from those of rH1N1, pBJ09 and Triple-1222 viruses (* is with *P*<0.05, ** is with *P*<0.01; ANOVA).

The viral distribution in nasal turbinates, trachea, lung, liver, spleen, kidney, and brain of mice were investigated. Apart from the nasal turbinates, trachea and lung, the presence of virus was not detected in the other tissues, revealing that all these viruses were restricted to the respiratory system of mice. In the nasal turbinates, rH1N1 virus replicated with significantly lower titers than pBJ09 virus (*P*<0.01) (Fig. 3*A*). As shown in Fig. 3*B and C*, the titers of rH1N1, pBJ09, and Triple-1222 viruses were higher than those of Eurasian-204 and hTJ15 viruses in trachea and lung on days 3, 5, and 7 p.i. (*P*<0.05) (except for hTJ15 in trachea on day 3 p.i. and Eurasian-204 in lung on day 5 p.i.).

Taken together, these results indicated that rH1N1 virus had similar infectivity and pathogenicity to that of pandemic H1N1/2009 and triple-reassortant viruses, while it had higher pathogenicity than the seasonal human H1N1 and Eurasian avian-like H1N1 viruses.

### Histopathology and immunohistochemistry of rH1N1 virus in mice

**Figure 4:**
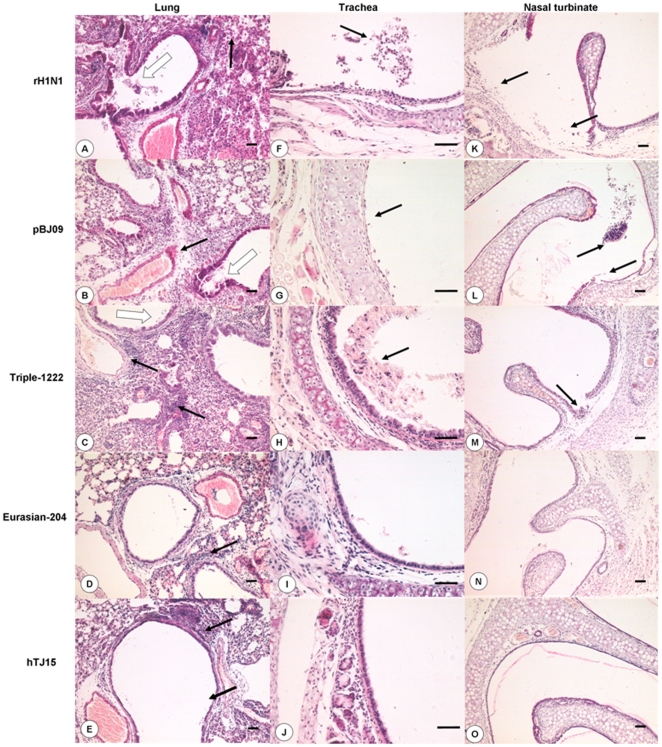
Histopathological changes in H1 virus-infected mice. Photomicrographs of H&E stained sections from nasal turbinates, trachea and lungs of mice on day 7 p.i. are shown. **(** ***A)*** **rH1N1 virus-infected lung.** Observation of extensive dropout and necrosis of mucous epithelium cells (open arrows), inflammatory cell infiltration and thickening of alveolar walls (solid arrows). **(** ***B*** **) pBJ09 virus-infected lung.** Dropout of mucous epithelium (open arrows), interstitial edema near the small blood vessels (solid arrows). **(** ***C*** **) Triple-1222 virus-infected lung.** Inflammatory cells infiltrate near the blood vessels (solid arrows) and dropout of mucous epithelium cells (open arrows). **(** ***D*** **) Eurasian-204 virus-infected lung.** Few inflammatory cells infiltrated near the bronchiole and blood vessels (solid arrows). **(** ***E*** **) hTJ15 virus-infected lung.** Few inflammatory cell infiltrated near the bronchiole and blood vessels (solid arrows). **(F) rH1N1 virus-infected trachea.** Dropout of mucous epithelium and inflammatory cell infiltration (solid arrows). **(** ***G*** **) pBJ09 virus-infected trachea.** Severe dropout of mucous epithelium cells in trachea, lamina propria bareness (solid arrows). **(** ***H*** **) Triple-1222 virus-infected trachea.** Dropout of mucous epithelium cells and many erythrocytes (solid arrows). **Eurasian-204 virus-infected trachea (** ***I*** **) and hTJ15 virus-infected trachea (J).** Rare histopathologic lesion. **(** ***K*** **) rH1N1 virus-infected nasal turbinate.** Dropout of mucous epithelium cells (solid arrows). **(** ***L*** **) pBJ09 virus-infected nasal turbinate.** Extensive dropout of mucous epithelium cells in nasal turbinates (solid arrows). **(** ***M*** **) Triple-1222 virus-infected nasal turbinate.** Lesional tunica mucosa nasi (solid arrows). **Eurasian-204 virus infected nasal turbinates (** ***N*** **) and hTJ15 virus-infected nasal turbinates (** ***O*** **).** No apparent histopathologic lesion. Bar = 50 µm.

To evaluate the histopathological changes between rH1N1 and other H1 viruses in mice, nasal turbinate, trachea, and lung samples from infected mice were fixed in 10% neutral buffered formalin and processed for routine histology. Representative histopathological changes are shown in Fig. 4. Lungs of all mice infected with viruses showed varying degrees of pneumonia. The histopathological changes in mice infected with rH1N1, pBJ09 and Triple-1222 viruses were more severe than those of other viruses. Severe bronchiolitis, peribronchiolitis, and bronchopneumonia were observed in mice infected with rH1N1, pBJ09, and Triple-1222 viruses. These changes were characterized by dropout and necrosis of mucous epithelium cells in the bronchioles, inflammatory cell infiltration near the small blood vessels, interstitial edema and thickening of the alveolar walls, and alveolar lumen flooded with dropout of alveolar cell, erythrocytes and inflammatory cells. Tunica mucosa tracheae and tunica mucosa nasi were severely damaged and a number of mucous epithelial cell exfoliates were found in the tracheal and nasal cavities. The Eurasian-204- and hTJ15-infected groups showed mild pathological lesions, which was characterized by inflammatory cell infiltration around the bronchioles and small blood vessels in lung, while no apparent histological changes were observed in the trachea and nasal turbinates.

Immunohistochemistry showed that viral antigen was distributed in deciduous alveolar cells, macrophages, infiltrative lymphocytes, lung bronchiolar epithelium cells, deciduous epithelial cells, tracheal infiltrative inflammatory cells, deciduous epithelium cells, and infiltrative inflammatory cells in nasal cavity of rH1N1, pBJ09 and Triple-1222 infected mice (Fig. S1). In the Eurasian-204 and hTJ15 infected mice, viral antigens were detected in the deciduous alveolar cells and mucosal epithelium cells in lung, but were rarely found in trachea and mucosal lamina propria cells of nasal turbinates.

In general, more severe histopathological changes were observed in the respiratory tract of mice infected with rH1N1, H1N1/2009, and triple-reassortant swine viruses than those infected with Eurasian avian-like and seasonal human influenza viruses.

### Transmissibility of rH1N1 virus in a guinea pig model

**Figure 5:**
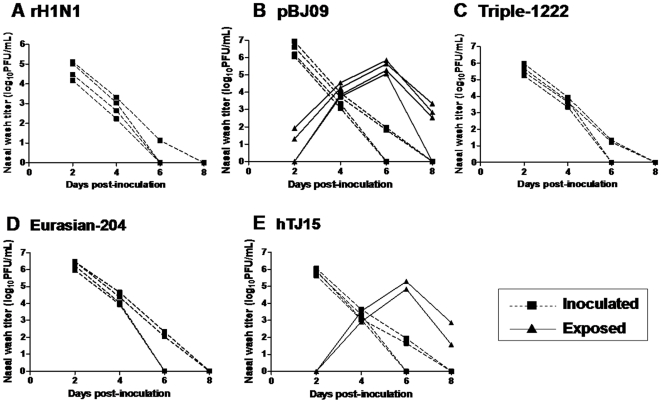
Contact transmission of rH1N1 (*A*), pBJ09 (*B*), Triple-1222 (*C*), Eurasian-204 (*D*) and hTJ15 (*E*) viruses in guinea pigs. Four guinea pigs were intranasally inoculated with 10^4.0^ PFU of virus and each animal was housed in a separate cage. At 24 h p.i., a naïve guinea pig was placed in the same cage with each inoculated guinea pig. Virus titers in nasal washes were plotted as a function of time post-inoculation. Titers of intranasally inoculated animals are represented by dashed lines and solid squares; virus titers of exposed guinea pigs are shown with solid lines and solid triangles. The limit of virus detection was 10 PFU/mL.

Efficient transmissibility is a typical feature of pandemic H1N1/2009 virus. To determine whether rH1N1 virus possesses this property, we evaluated the contact transmissibility of rH1N1 virus in guinea pigs. Simultaneously, H1N1/2009, triple-reassortant swine, Eurasian avian-like swine, and seasonal human influenza viruses were also evaluated for their transmissibility in guinea pigs. Transmission was determined by the detection of virus in the nasal washes and seroconversion. The animal experiments showed that no obvious clinical signs were observed in any of the guinea pigs including inoculated and un-inoculated animals during the observation period. All the tested viruses were able to replicate in the nasal passages of inoculated animals, with peak titers from 4.7–6.5 log_10_ PFU/mL. As shown in Fig. 5, no virus shedding or seroconversion was detected in any exposed animals of rH1N1 groups, as well as Triple-1222 and Eurasian-204 groups. By contrast, pBJ09 virus showed efficient transmission that all four exposed animals were detected to be positive, while hTJ15 transmitted less efficiently than pBJ09 with two of four exposed animal becoming infected. These results indicated that although rH1N1 virus has a pathogenicity that is similar to H1N1/2009 virus in mice, it does not acquire the transmissibility in guinea pigs which is a typical trait of H1N1/2009 virus.

### Identification of the gene segment contributing to the different transmissibility between rH1N1 and H1N1/2009 viruses

To determine which gene segment contributes to the transmission difference between rH1N1 and pBJ09 viruses, reverse genetics was used to construct a series of viruses with the gene segments from rH1N1 and pBJ09 viruses and their transmissibility were tested in guinea pigs. Two strategies of virus constructing were approached. First, to determine whether the surface genes or the internal genes contribute to the different transmissibility between rH1N1 and pBJ09 viruses, two reassortants were constructed by introducing two surface genes and six internal genes from pBJ09 in the rH1N1 backbone, respectively. As shown in Table 2, the novel reassortant virus, pBJ09HANA:rH1N1, transmitted as efficiently among guinea pigs as pBJ09 virus, with 100% efficiency; while the rH1N1HANA:pBJ09 virus transmitted inefficiently, with only one of four exposed animals becoming infected. The results indicated that the surface genes of pBJ09 played an important role in efficient transmissibility and that the internal genes were also involved in the transmission. To further identify which single gene was related to the transmissibility, a series of reassortants with individual genes from pBJ09 using the rH1N1 backbone were rescued and their transmissibility were investigated in guinea pigs. The transmission experiments showed that only the reassortants with HA or NS genes replaced by that of pBJ09 in the backbone of rH1N1 could transmit between guinea pigs, but the respective transmission efficiency was lower than that of pBJ09 virus (Table 2). The pBJ09HA: rH1N1 virus transmitted to three of four exposed animals, while pBJ09NS: rH1N1 virus transmitted to two of four exposed animals. It is noteworthy that pBJ09HA: rH1N1 virus replicated less efficiently in the nasal wash than those of pBJ09 and rH1N1 viruses. However, the replication ability was increased by the introduction of both HA and NA genes from pBJ09 in the rH1N1 backbone (*P*<0.01). Collectively, the present findings demonstrated that the HA and NS genes of H1N1/2009 virus contribute to viral transmissibility, but are not sufficient for efficient transmission. The HA gene of H1N1/2009 virus, when combined with H1N1/2009 NA, confers efficient contact transmission in guinea pigs.

**Table 2:** Direct contact transmission of wild-type and rH1N1-pBJ09 reassortant viruses in guinea pigs.

	Inoculated animals		Contact animals		
Virus	No. with virus detection (peak titer)^a^	No. with seroconversion (HI titer range)^b^	No. with virus detection (peak titer)^a^	No. with seroconversion (HI titer range)^b^	Transmission
pBJ09	4/4 (6.5±0.4)	4/4 (320)	4/4 (5.5±0.3)	4/4 (160–320)	Efficient
rH1N1	4/4 (4.7±0.4)	4/4 (40–80)	0/4	0/4	None
pBJ09HANA:rH1N1	4/4 (5.9±0.6)	4/4 (160–320)	4/4 (5.8±0.8)	4/4 (80–320)	Efficient
rH1N1HANA:pBJ09	4/4 (5.3±0.2)	4/4 (80–160)	1/4 (3.1)	1/4 (40)	Inefficient
pBJ09HA: rH1N1	4/4 (4.4±0.4)	4/4 (80–160)	3/4 (3.8±0.7)	3/4 (40–80)	Inefficient
pBJ09NA: rH1N1	4/4 (5.0±0.3)	4/4 (80)	0/4	0/4	None
pBJ09PB2: rH1N1	4/4 (4.0±0.3)	4/4 (40–80)	0/4	0/4	None
pBJ09PB1: rH1N1	4/4 (4.8±0.4)	4/4 (80)	0/4	0/4	None
pBJ09PA:rH1N1	4/4 (5.0±0.5)	4/4 (80)	0/4	0/4	None
pBJ09NP: rH1N1	4/4 (4.4±0.2)	4/4 (40)	0/4	0/4	None
pBJ09M: rH1N1	4/4 (5.3±0.5)	4/4 (40–80)	0/4	0/4	None
pBJ09NS: rH1N1	4/4 (6.0±0.6)	4/4 (40–80)	2/4 (4.6±0.2)	2/4 (40–80)	Inefficient

a Number of the infected guinea pigs/total number of the guinea pigs (mean peak titer log_10_PFU/mL ± standard deviation).

b Serum was collected on day 18 p.i. and seroconversion was confirmed by hemagglutination inhibition (HI) assay.

### Comparison of the amino acid of HA and NS proteins between rH1N1 and pBJ09 viruses

The above data proved that the HA and NS genes contributed to the H1N1/2009 viral transmissibility. To further determine the molecular basis of the transmissibility, we compared the deduced amino acid sequences of the HA and NS proteins between rH1N1 and pBJ09 viruses. The comparison of amino acid sequences showed that there were a total of 25 amino acids in HA protein, 20 amino acids in NS1 protein, and 8 amino acids in NS2 protein differences between rH1N1 and pBJ09 viruses, suggesting that these amino acids might play an important role in the transmissibility (Table 3, Table S1, Table S2). Receptor binding specificity plays an important role in the transmission of influenza virus [17]. It is noteworthy that four amino acid sites, D135M, R136K, T219I, and A227E, located in the HA receptor binding site, might partially contribute to the different transmission of these two virus in guinea pigs.

**Table 3:** Amino acid differences of the HA proteins between rH1N1 and pBJ09 viruses^a^.

	Amino acid position in HA^b^																								
Virus	79	92	112	127	135	136	146	149	171	206	212	219	227	259	260	263	273	300	316	323	347	358	376	445	475
rH1N1	F	N	E	N	**D**	**R**	G	R	N	S	K	**T**	**A**	I	K	S	S	V	M	I	I	D	G	M	D
pBJ09	S	S	D	S	**M**	**K**	S	K	D	T	T	**I**	**E**	M	E	A	P	I	L	V	V	E	K	K	N

a Amino acids in the HA receptor binding site are bold.

b H3 numbering.

## Discussion

The historic 1957 and 1968 pandemic influenza viruses were considered to be reassortant viruses that originated from avian and human influenza viruses [18]–[20]. The current 2009 pandemic virus, a swine-origin H1N1 influenza virus, was a more complicated reassortant derivative of avian, swine, and human viruses [6], [7]. Although pandemic influenza viruses are usually generated through reassortment, it is not clear whether the basic reassortment without amino acid mutation of viral genome could result in the emergence of a pandemic virus. In the present study, we artificially engineered a reassortant influenza virus (rH1N1) with the same genotype as H1N1/2009 virus by reverse genetics and evaluated the pathogenicity in mice and transmissibility in guinea pigs. Our results showed that the pathogenicity of rH1N1 virus in mice was similar to that of H1N1/2009 virus, while rH1N1 virus did not possess the transmissibility among guinea pigs as H1N1/2009 virus. Furthermore, we demonstrated that HA and NS genes were crucial for transmissibility of H1N1/2009 virus, and the HA gene combining with the H1N1/2009 NA confers efficient transmission.

Previous studies showed that H1N1/2009 viruses replicated efficiently and caused more severe pathological lesions in mice, ferrets, and non-human primates than the circulating seasonal human H1N1 [13]. Although rH1N1 was a reassortant derived from the triple-reassortant H1N2 and Eurasian avian-like swine H1N1 viruses, its characteristics *in vitro* and *in vivo* were similar to those of the triple-reassortant, while being significantly different from Eurasian avian-like. The current study performed in mice indicated that rH1N1, pBJ09, and Triple-1222 viruses induced more severe clinical signs, and replicated more efficiently in the trachea and lung of mice than Eurasian swine and seasonal human viruses. rH1N1, pBJ09, and Triple-1222 viruses induced higher mortality and more severe histopathological changes in the respiratory system than other viruses. The high pathogenicity of Triple-1222 virus was consistent with the results of triple-reassortant H1N1 swine influenza isolated in humans [21]. However, the pathogenicity of pBJ09 (LD_50_ = 10^3.6^ PFU) was higher than those of H1N1/2009 influenza viruses (LD_50_≥10^6.0^ PFU), which were avirulent in mice in previous studies [13], [21]–[23]; except for the WSLH34936 virus (LD_50_ = 10^4.5^ PFU) [13]. The present results indicated that the low pathogenicity observed in mice was not a common feature of H1N1/2009 viruses, and that the pathogenicity of rH1N1 virus was similar to (or higher than) that of H1N1/2009 virus. Comparing the amino acid sequences of pBJ09 virus with those of H1N1/2009, several genetic differences were found between pBJ09 virus and the previously isolated strains. pBJ09 virus shared some mutations in common with the pandemic variants that were predominant in the southern hemisphere in the winter of 2010, such as PB2 K660R, PB1 T257A, HA E391K, and NS1 M93I mutations [24]. However, it remains unknown whether these genetic changes contribute to the higher virulence and so need further investigation.

H1N1/2009 influenza virus efficiently transmitted in humans. Transmission animal models, including guinea pigs, pigs, and ferrets, have been established [13], [25]–[27]. Our guinea pig model experiments indicated that H1N1/2009 virus had efficient transmissibility among guinea pigs *via* a direct contact route. However, like Triple-1222 and Eurasian-204 swine viruses, rH1N1 virus did not transmitted in this animal model. To investigate which gene segment contributed to the different transmissibility between rH1N1 and pandemic H1N1/2009 viruses, we constructed a series of reassortants of these viruses and evaluated their transmissibility. The results showed that rH1N1 virus was transmissible among guinea pigs after acquiring the HA or NS gene from H1N1/2009 virus. The role of the HA gene in transmission has been previously demonstrated [28]–[31]. We also found that introduction of single HA gene from H1N1/2009 virus resulted in inefficient replication and transmission of reassortant in guinea pigs. By contrast, the H1N1/2009 HA gene when combined with the H1N1/2009 NA gene conferred both efficient replication and contact transmission between guinea pigs. These results suggest that the interaction of HA and NA genes of H1N1/2009 virus affected viral replication and transmission. The NS gene has been demonstrated to be relevant to viral replication and pathogenicity [32]–[34]. In the present study, the replication of the rH1N1 virus in the nasal cavity of guinea pigs increased significantly after the replacement of the NS gene by those of H1N1/2009 (*P*<0.01), which is a plausible in terms of the transmissibility of the pBJ09NS:rH1N1 virus among guinea pigs. However, some reassortants with high titers were unable to transmit among guinea pigs. Additionally, viruses (pBJ09 and pBJ09HANA:rH1N1) which can efficient transmit among guinea pigs had high peak virus titers in the inoculated animals. Howerver, pBJ09NS:rH1N1 with comparable peak virus titers to pBJ09HANA:rH1N1 (P>0.05) transmitted inefficient among guinea pigs. These results indicated that high virus load of inoculated animal might played an important role in the transmission, but other factor(s) also contribute to the transmissibility of influenza viruses. Further molecular analysis of HA, NS1, and NS2 proteins showed that total of 53 amino acid differences between rH1N1 and pBJ09 might be involved in the transmissibility difference. The affinity of the viral HA protein for sialic acid α-2,6 linked glycan confers transmission of the 1918 H1N1 influenza virus in ferrets (40). Four amino acid differences (D135M, R136K, T219I and A227E) occurred in the HA receptor binding site, which might play an important role in viral transmissibility. Recently, Haan et al reported that only two residues (A200T and A227E) were responsible for the dramatic receptor binding change from swine to pandemic H1 hemagglutinin [35]. rH1N1 possessed 227A on HA protein, while pBJ09 had 227E. Further study is warranted to determine whether this substitution contributed to the different transmissibility between rH1N1 and pBJ09 viruses. Nevertheless, these results demonstrated that the reassortment between triple-reassortant and Eurasian avian-like swine viruses is not sufficient for transmissibility as H1N1/2009 virus, and further mutations may be necessary.

In summary, we demonstrated that infectivity, replication, and pathogenicity of rH1N1, a virus with same gene combination as H1N1/2009 virus, are similar to those of the pBJ09 (H1N1/2009) virus. However, transmissibility in guinea pigs of rH1N1 virus was not found. Moreover, we also demonstrated that the HA and NS genes contributed to the transmission of H1N1/2009 virus; and HA gene of H1N1/2009 virus, when combined with the H1N1/2009 NA gene, conferred efficient contact transmission among guinea pigs. The present findings demonstrated that a virus with the same gene combination as H1N1/2009 virus did not directly acquire the transmission phenotype as H1N1/2009 virus, indicating that additional variation was needed for the efficient transmissibility.

## Materials and Methods

### Ethics statement

All animal research was approved by the Beijing Association for Science and Technology, the approve ID is SYXK (Beijing) 2007-0023, and complied with the guidelines of Beijing laboratory animal welfare and ethical of Beijing Administration Committee of Laboratory Animals.

### Viruses

The North American triple-reassortant swine H1N2 influenza virus, A/swine/Guangdong/1222/2006 (Triple-1222), was originally isolated from diseased pigs in 10-day old embryonated chicken eggs and passaged twice in MDCK cells [8]. Eurasian avian-like H1N1 swine virus, A/swine/Fujian/204/2007 (Eurasian-204), was isolated from healthy pigs in 10-day old embryonated chicken eggs and passaged twice in MDCK cells [16]. The pandemic H1N1/2009 influenza virus, A/Beijing/7/2009 (pBJ09), was isolated from a young patient with an influenza-like illness in December 2009 in 10-day old embryonated chicken eggs and passaged once in MDCK cells. Seasonal human H1N1 influenza virus A/Tianjin/15/2009 (hTJ15) was kindly provided by the Chinese Centers for Disease Control (CDC).

### Generation of reassortant viruses by reverse genetics

Reverse transcription-PCR (RT-PCR) amplicons of the viral genes were cloned into a dual-promoter plasmid, PHW2000. MDCK and 293T cells were co-cultured and transfected with 0.5 µg of each of the eight plasmids and 10 µL lipofectamine 2000 (Invitrogen) in a total volume of 1 mL of Opti-MEM (Invitrogen). After incubation at 37 °C for 6 h, the transfection mixture was removed from the cells and 2 mL of Opti-MEM containing 1 µg/mL of TPCK-trypsin was added. After 72 h, the supernatant was inoculated in MDCK cells to produce stock viruses. Viral RNA was extracted and analyzed by RT-PCR, and each viral segment was sequenced to confirm the identity of the virus. The titers of the stock viruses were determined by plaque assay on MDCK cells. All experiments with live viruses and transfectants generated by reverse genetics were performed in a biosafety level 3 containment laboratory approved by Ministry of Agriculture of the People's Republic of China and the approve ID is CNAS BL0017.

### Viral growth kinetics

MDCK cells were infected with each virus at a multiplicity of infection (MOI) of 0.05, overlaid with serum-free Dulbecco's Modified Eagle Medium (DMEM) (Gibco, USA) containing 2.5 µg/mL N-acetyl-trypsin (Sigma–Aldrich, USA) and incubated at 37°C. Cell supernatants were harvested every 12 h until 72 h post-infection (p.i.), and titrated by plaque assays.

### Viral pathogenicity in mice

Six-week-old female BALB/c mice (Vital River Laboratories [VRL], China) were anaesthetized with Zoletil (tiletamine-zolazepam; Virbac S.A., Carros, France; 20 µg/g) and intranasally inoculated with 50 µL of infectious virus diluted in sterile phosphate-buffered saline (PBS). The 50% mouse infectious dose (MID_50_) and 50% lethal dose (LD_50_) were determined as previously described [36], [37]. Briefly, mice were euthanized on day 3 p.i., and homogenized lung tissues were obtained to determine the MID_50_ calculated by the method of Reed and Muench [38]. Four mice per virus were monitored daily for 14 days p.i. for morbidity as measured by weight loss, and mortality to determine the LD_50_. Any mouse that lost >30% of its pre-inoculation body weight was euthanized. Three mice inoculated with 10^4.0^ plaque forming units (PFU) of each virus were euthanized on days 3, 5 and 7 p.i. and the nasal turbinates, trachea, lung, liver, spleen, kidney and brain were collected for virus detection and titration.

### Histopathology and immunohistochemistry (IHC) in mice

A portion of the nasal turbinates, trachea and lung of euthanized mice were preserved in 10% phosphate-buffered formalin on day 7 p.i. Samples were then processed for paraffin embedding and cut into 5-µm thick sections. One section from each tissue sample was stained with hematoxylin and eosin (H&E) stain. Another section was processed for immunohistological staining with a mouse monoclonal antibody (AA5H, Abcam, Hong Kong) specifically against influenza A virus NP and a goat-anti-mouse IgG biotin conjugated affinity purified antibody (Chemicon, USA). Specific antigen–antibody reactions, substrate and chromogen were visualized by brown staining using diaminobenzidine-tetrahydrochloride (DAB) (Sigma, USA), then hematoxylin was used as a counterstain.

### Contact transmission in guinea pigs

Hartley strain female SPF/VAF guinea pigs weighing between 300–350 g, which were seronegative for influenza virus were obtained from Vital River Laboratories (VRL) in China. Guinea pigs were anesthetized by intramuscular injection of Zoletil 100 (tiletamine-zolazepam; Virbac, France; 10–15 mg/kg) prior to all procedures including inoculation, nasal wash collection, collection of blood). Four guinea pigs were inoculated intranasally with 10^4.0^ PFU tested virus in 300 µL PBS. Each infected animal was placed in the same cage with one naïve guinea pig at 24 h p.i.. Nasal washes were collected from all eight guinea pigs on days 2, 4, 6 and 8 p.i.

### Statistical analyses

Statistically significant differences between experimental groups were determined using of the analysis of variance (ANOVA) method. A *P*-value <0.05 was considered statistically significant.

## Supporting Information

Figure S1 **Immunohistochemical analysis of virus-infected mice.** Tissue sections of the nasal turbinates trachea and lungs were stained with a monoclonal antibody against influenza A virus Nucleoprotein (AA5H); made visible by brown staining. Data taken on day 7 p.i.. **(** ***A*** **) rH1N1 virus-infected lung.** Viral antigen staining in alveolar cells and lymphocyte in the lung (arrows). **(** ***B*** **) pBJ09 virus-infected lung.** Infected bronchiolar mucosal epithelium cells in the lung (arrows). **(** ***C*** **) Triple-1222 virus-infected lung.** Infected deciduous mucosal epithelium cells in the lungs (arrows). **(** ***D*** **) Eurasian-204 virus-infected lung.** Rare virus-infected deciduous alveolar cells (arrows). **(** ***E*** **) hTJ15 virus-infected lung.** Infected alveolar cells in the lungs (arrows). **(** ***F*** **) rH1N1 virus-infected trachea.** A number of infected deciduous mucosal epithelium cells in the trachea (arrows). **(** ***G*** **) pBJ09 virus-infected trachea.** Infected mucosal epithelium cells in the trachea (arrows). **(** ***H*** **) Triple-1222 virus-infected trachea.** Weak viral antigen staining in mucosal epithelium cells in the trachea (arrows). **(** ***I*** **) Eurasian-204 virus-infected trachea.** No positive viral antigen staining in the trachea. **(** ***J*** **) hTJ15 virus-infected trachea.** Rare viral antigen staining in the mucosal epithelium cells in the trachea (arrows). **(** ***K*** **) rH1N1 virus-infected nasal turbinate.** Infected mucosal epithelium cells in the nasal turbinates (arrows). **(** ***L*** **) pBJ09 virus-infected nasal turbinate.** Infected deciduous mucosal epithelium cells in the nasal turbinates (arrows). **(** ***M*** **) Triple-1222 virus-infected nasal turbinate.** Infected serous gland epithelium cells in the nasal turbinates (arrows). **(** ***N*** **) Eurasian-204 virus-infected nasal turbinate.** Less infected cells in the lamina propria of the nasal turbinates (arrows). **(** ***O*** **) hTJ15 virus-infected nasal turbinate.** Less infected cells in the lamina propria of the nasal turbinates (arrows). Bar = 50 µm.
(TIF)

Table S1 **Amino acid differences of the NS1 proteins between rH1N1 and pBJ09 viruses.**
(PDF)

Table S2 **Amino acid differences of the NS2 proteins between rH1N1 and pBJ09 viruses.**
(PDF)
