# Supplementary figures and images for: Characterization of an Artificial Swine-Origin Influenza Virus with the Same Gene Combination as H1N1/2009 Virus: A Genesis Clue of Pandemic Strain
Source: PLoS One. 2011 Jul 25;6(7):e22091. doi: 10.1371/journal.pone.0022091 (PMC3143117; doi:10.1371/journal.pone.0022091)

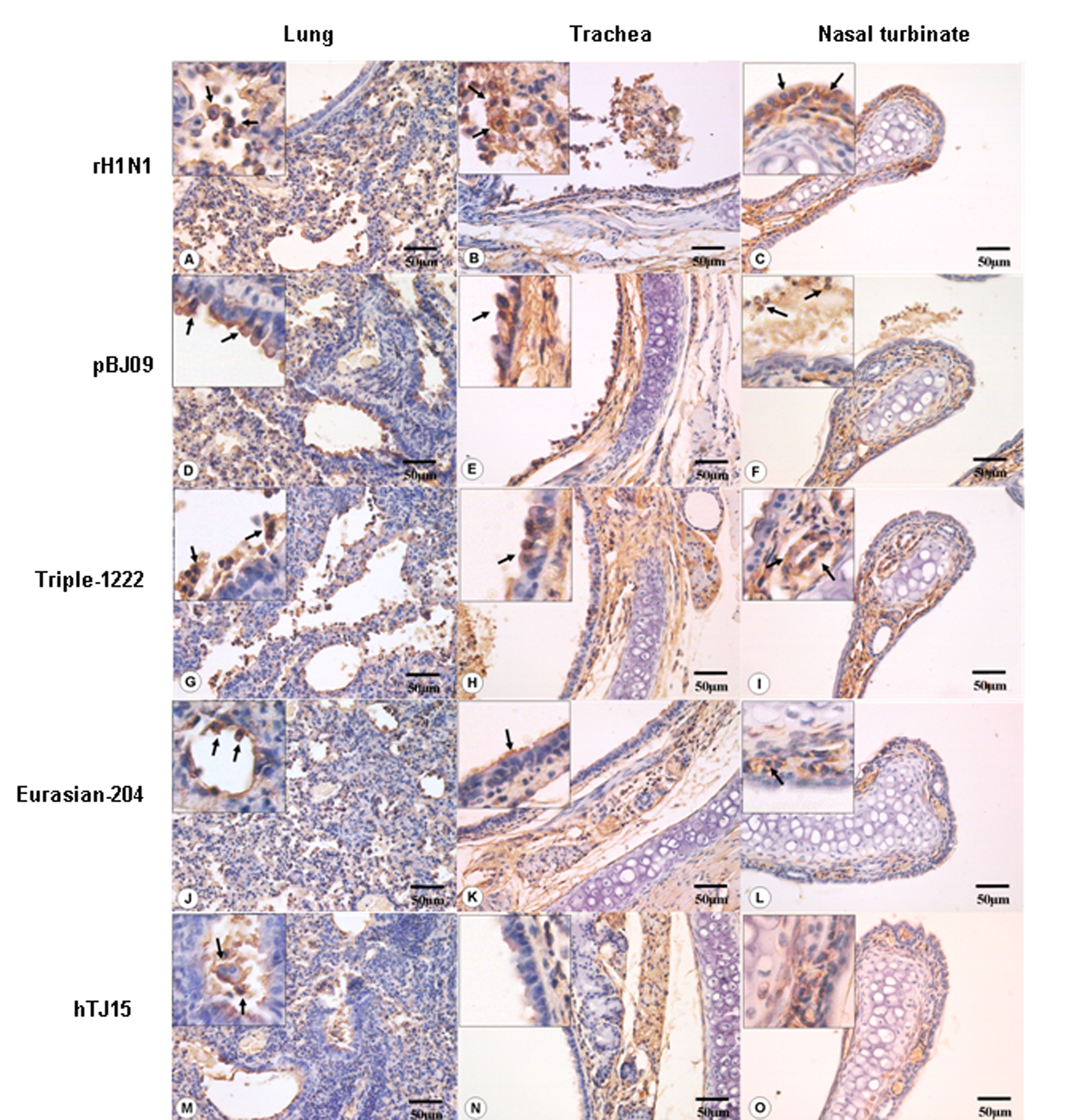

Supplement: Figure S1 — Immunohistochemical analysis of virus-infected mice. Tissue sections of the nasal turbinates trachea and lungs were stained with a monoclonal antibody against influenza A virus Nucleoprotein (AA5H); made visible by brown staining. Data taken on day 7 p.i.. ( A ) rH1N1 virus-infected lung. Viral antigen staining in alveolar cells and lymphocyte in the lung (arrows). ( B ) pBJ09 virus-infected lung. Infected bronchiolar mucosal epithelium cells in the lung (arrows). ( C ) Triple-1222 virus-infected lung. Infected deciduous mucosal epithelium cells in the lungs (arrows). ( D ) Eurasian-204 virus-infected lung. Rare virus-infected deciduous alveolar cells (arrows). ( E ) hTJ15 virus-infected lung. Infected alveolar cells in the lungs (arrows). ( F ) rH1N1 virus-infected trachea. A number of infected deciduous mucosal epithelium cells in the trachea (arrows). ( G ) pBJ09 virus-infected trachea. Infected mucosal epithelium cells in the trachea (arrows). ( H ) Triple-1222 virus-infected trachea. Weak viral antigen staining in mucosal epithelium cells in the trachea (arrows). ( I ) Eurasian-204 virus-infected trachea. No positive viral antigen staining in the trachea. ( J ) hTJ15 virus-infected trachea. Rare viral antigen staining in the mucosal epithelium cells in the trachea (arrows). ( K ) rH1N1 virus-infected nasal turbinate. Infected mucosal epithelium cells in the nasal turbinates (arrows). ( L ) pBJ09 virus-infected nasal turbinate. Infected deciduous mucosal epithelium cells in the nasal turbinates (arrows). ( M ) Triple-1222 virus-infected nasal turbinate. Infected serous gland epithelium cells in the nasal turbinates (arrows). ( N ) Eurasian-204 virus-infected nasal turbinate. Less infected cells in the lamina propria of the nasal turbinates (arrows). ( O ) hTJ15 virus-infected nasal turbinate. Less infected cells in the lamina propria of the nasal turbinates (arrows). Bar = 50 µm. (TIF) [file pone.0022091.s001.tif]
