# Supplementary material for: Characterization of an Artificial Swine-Origin Influenza Virus with the Same Gene Combination as H1N1/2009 Virus: A Genesis Clue of Pandemic Strain
Source: PLoS One. 2011 Jul 25;6(7):e22091. doi: 10.1371/journal.pone.0022091 (PMC3143117; doi:10.1371/journal.pone.0022091)
Supplement: Table S1 — Amino acid differences of the NS1 proteins between rH1N1 and pBJ09 viruses. (PDF) [file pone.0022091.s002.pdf]

Table S1. Amino acid differences of the NS1 proteins between rH1N1 and pBJ09 viruses

Virus	Amino acid position in NS1																			
	7	78	91	93	114	118	123	129	137	139	145	153	171	172	197	198	206	207	209	211
rH1N1	V	K	A	M	S	M	I	I	T	D	V	G	D	I	I	L	R	N	D	R
pBJ09	M	R	S	I	P	L	V	V	I	N	I	E	Y	V	N	I	C	D	N	K
