# Supplementary material for: Characterization of an Artificial Swine-Origin Influenza Virus with the Same Gene Combination as H1N1/2009 Virus: A Genesis Clue of Pandemic Strain
Source: PLoS One. 2011 Jul 25;6(7):e22091. doi: 10.1371/journal.pone.0022091 (PMC3143117; doi:10.1371/journal.pone.0022091)
Supplement: Table S2 — Amino acid differences of the NS2 proteins between rH1N1 and pBJ09 viruses. (PDF) [file pone.0022091.s003.pdf]

Table S2. Amino acid differences of the NS2 proteins between rH1N1 and pBJ09 viruses

	Amino acid position in NS2							
Virus	6	40	52	54	80	83	92	115
rH1N1	M	F	T	D	T	V	S	T
pBJ09	V	I	M	N	I	M	N	A
